# Distributional dynamics of child mortality in Africa: quantile evidence from economic, environmental, and demographic transitions

**DOI:** 10.3389/fpubh.2026.1730385

**Published:** 2026-02-18

**Authors:** Mohammad Ridwan, Jeremy Ko, Zulfiquar Ali Antor, Hossein Ali Fakher, Afsana Akther, Chun Kai Leung, Arsene Mouongue Kelly, Wai Kit Ming

**Affiliations:** 1 Department of Economics, Noakhali Science and Technology University, Noakhali, Bangladesh; 2 Center for Comparative and International Studies, ETH, Zurich, Zurich, Switzerland; 3 Department of Business Management, Ayandegan Institute of Higher Education, Tonekabon, Iran; 4 Global Society and Sustainability Lab., The University of Hong Kong, Pok Fu Lam, Hong Kong SAR, China; 5 Dschang School of Economics and Management, University of Dschang, Dschang, Cameroon; 6 Department of Infectious Diseases and Public Health, City University of Hong Kong, Kowloon, Hong Kong SAR, China

**Keywords:** Africa, air pollution, child mortality, fertility rate, health expenditure, sustainable development

## Abstract

Child mortality remains one of Africa’s most persistent development challenges, undermining progress toward Sustainable Development Goal (SDG) 3.2, while its economic, environmental, and demographic correlates also intersect with SDGs 1, 2, 11, and 13. Although previous studies have examined mortality trends, limited attention has been devoted to understanding how economic growth, air pollution, health expenditure, urbanization, and fertility interact asymmetrically across the mortality distribution, a gap that restricts policy from targeting vulnerability where it is most pronounced. In an attempt to fill this gap and provide actionable solutions, the present study investigates the distributional dynamics of child mortality in 53 African countries from 2000 to 2024 using the method-of-moments quantile regression framework adjusted for cross-sectional dependence and slope heterogeneity and supported by Driscoll-Kraay and system-GMM estimators for robustness. The findings show that economic growth, higher public health spending, and urbanization are strongly associated with reductions in under-five deaths, particularly in high-mortality countries, whereas air pollution and fertility significantly increase mortality, with fertility emerging as the dominant risk factor. These results underscore the heterogeneous nature of mortality drivers across levels of vulnerability, highlighting that effective intervention requires tailored and multidimensional strategies. The study provides evidence-based policy recommendations to support targeted reforms that accelerate child survival and sustainable development across Africa.

## 1 Introduction

Child mortality is a significant health and development issue worldwide and exemplifies the continued disparities in accessing healthcare and living conditions, as well as economic opportunities. Although the world has experienced a significant reduction in under-five mortality since the first years of the century, mortality rates in African countries remain several times greater than those in high-income countries, which negatively affects the achievement of Sustainable Development Goal (SDG) 3.2 (1). Although the world’s development has accelerated, the situation in sub-Saharan Africa continues to have the highest infant and child death rates, which constitute a disproportionate percentage of global deaths (2, 3). The fact that these disparities still exist highlights the fact that the regions are still struggling to attain equity in child survival. Low child mortality rates not only limit socioeconomic development through the loss of human capital but also reinforce poverty and burden weak health systems (4). Therefore, knowledge of the multidimensional factors that contribute to child mortality in Africa is crucial not only for achieving SDG 3.2 but also for enabling the rest of the developmental and demographic transitions on the continent.

Child mortality determinants are multidimensional, spanning economic, environmental, and demographic dimensions that influence health outcomes. Economic performance is correlated with better child survival, as higher income leads to more access to nutrition, education, and medical services (5, 6). The advantages of these changes vary between income groups, and the ability of economic development to mitigate mortality differs between low- and high-income economies (4, 7). Environmental degradation, especially air pollution, is another decisive factor. Research in Asia, Africa, and Europe reports that high levels of particulate matter and carbon emissions increase infant and child death in resource-intensive environments (8–11). Demographic variables such as fertility and urbanization are significant. While urbanization may improve healthcare and sanitation, high population growth remains a prominent risk factor, increasing death rates and restricting progress in most African countries (12–14). These forces are interconnected, indicating the complexity of mortality outcomes and the need for integrated analytical methods.

While researchers have studied isolated relationships between economic performance, fertility or environmental degradation and child mortality, they have not examined how these forces interact to determine survival outcomes in Africa. The interaction between fertility pressure and air pollution as key stressors in low-income environments and the effects of economic growth and health investment on reducing child deaths remain understudied (2, 15). Studies provide limited insight into whether the mortality-reducing benefits of income or urbanization persist when fertility and pollution increase simultaneously or if such relationships vary between high- and low-mortality nations (4). No study has examined how population increases, environmental degradation and health financing interact in African Union policy across continents. This study examines, for the first time, the joint effects of economic growth, health expenditures, urbanization, fertility, and air pollution on child mortality in Africa, providing new empirical knowledge of how developmental determinants of child survival in Africa are transformed by interactive and conditional effects previously concealed.

On the basis of these gaps, this research is expected to contribute to the overall knowledge of the interplay of economic, environmental, and demographic factors on child mortality in African Union member countries. The main hypothesis is to determine whether economic growth, increases in health spending and urbanization development advantages are mediated or counterbalanced by fertility strains and environmental deterioration. In particular, this study aims to determine whether there is an increase in the mortality risk of fertility and air pollution in even nations with economic development and growth in terms of sector health and how these impacts vary in the context of low or high mortality. Therefore, the research questions to be answered by this study are as follows: (1) How do economic growth, air pollution, health expenditure, urbanization and fertility jointly influence child mortality outcomes in African Union countries? (2) Are the effects of these determinants different at varying levels of mortality intensity, and an asymmetric or conditional effect is generated? (3) To what extent may the effects of fertility and pollution on mortality be reduced by better health spending and urbanization? The response to these questions will help the study provide an integrated and policy-relevant perspective on the mechanisms through which economic and demographic transitions influence child survival in Africa.

Building on and expanding the studies of Rahman et al. (1) and Fotio et al. (16), this study addresses several unresolved gaps in the child mortality literature for Africa. The present study situates child mortality within a multidimensional system of economic, environmental, and demographic transitions, and does so for a near-continental sample of 53 African countries over the 2000–2024 period, a region that continues to record the world’s highest under-five mortality rates and slower progress toward SDG 3.2 despite global declines. By explicitly modeling how growth, air pollution, health expenditure, urbanization, and fertility interact across the mortality distribution, the study uncovers vulnerabilities in high-mortality settings that are masked by conventional mean-based approaches and complements recent evidence on social determinants of under-five mortality in Africa.

Methodologically, the study contributes by applying the method-of-moments quantile regression framework of Machado and Silva (17), rooted in the quantile regression tradition of Koenker and Bassett (18), to a dynamic panel that is explicitly adjusted for cross-sectional dependence and slope heterogeneity, and by corroborating the results with Driscoll–Kraay standard errors and system-GMM estimators. This distribution-sensitive strategy shows that economic growth, higher public health spending, and urbanization are most mortality-reducing at the upper quantiles, while air pollution and fertility exert increasingly harmful effects toward the top of the distribution, with fertility emerging as the dominant driver of risk. In doing so, the study extends prior macro-panel work on economic, environmental, and institutional determinants of child mortality in Africa by revealing how their impacts vary across the spectrum of vulnerability. The findings therefore contribute both to the literature on child mortality, environmental health, and demographic transition in low-income settings, and to broader efforts to achieve SDG 3.2 on ending preventable under-five deaths, alongside related goals on poverty, urban sustainability, and climate action, in an African context increasingly characterized by rapid urbanization, environmental stress, and evolving population structures.

The remainder of this paper is organized as follows: Section 2 reviews the relevant literature and theoretical foundations. Section 3 details the data, variables, and empirical methodology. Section 4 presents and discusses the empirical results along with robustness analyses. Finally, Section 5 concludes with key findings, policy implications, and directions for future research on child survival and sustainable development in Africa.

## 2 Literature review and hypothesis development

The determinants of child mortality cannot be viewed in a unidimensional manner because of economic, environmental, and demographic factors, which must be combined in a multidimensional approach. This section reviews the available theoretical and empirical literature on the important mechanisms by which these factors influence mortality results, especially in the context of the African setting. It also highlights discrepancies in the previous results and presents testable hypotheses illustrating the asymmetric and interactive character of these dependent relationships. The discourse is thematic since it focuses on the economic, environmental, and demographic causes of child mortality.

### 2.1 Economic growth, health expenditure, and child mortality

Economic development and healthcare expenditure are two correlated trends that determine child survival in Africa. The increase in economic output will enhance income, nutrition, and educational and medical services, which subsequently will reduce child mortality (19, 20). Nevertheless, the extent of this advantage is determined by the fairness of income gains distribution and the availability of growth dividends to vulnerable groups, which is supported by evidence on the significance of inclusive development (21). Health spending, in its turn, has a greater impact on child survival by investing in infrastructure, preventive health, and service delivery infrastructure (22, 23). However, the impact of such spending can be minimized in case of mismanagement of the public resources or institutional inefficiency (24). All of these results suggest that as economies grow, mortality can be indirectly decreased, but in the long term, it is health investment that can be highly effective and efficient only when it is well-governed. Overall, sustainable reductions in child mortality require an integrated approach in which economic growth is complemented by well-governed and efficiently financed health systems. Hence, we would expect that both economic progress and health expenditure play crucial roles in improving child survival outcomes across African countries.

### 2.2 Environmental degradation, air pollution and child mortality

One of the most chronic dangers to child survival is environmental degradation especially in third world countries where rate of urbanization and loose regulatory measures are the leading cause of high rates of pollution. Carbon emissions and air pollution in particular are detrimental and put pressure on respiration, weak birth weight, and premature death (10, 11). Use of biomass fuel, poor consumption of energy and congestion in urban areas are other factors that expose children to toxic pollutants in African contexts (8). Poverty is also strengthened by environmental stress, which restricts access to nutrition and healthcare, as well as increases health susceptibility in infants and young children (9). It has been indicated that the trend to improve the air quality and climate change can offset the anticipated health benefits with regards to economic growth even (15). Therefore, environmental degradation—particularly air pollution—must be understood not merely as an ecological concern but as a critical public-health challenge that undermines sustainable development and endangers child survival. Hence, we hypothesize that higher levels of air pollution are associated with increased child mortality across African countries.

### 2.3 Fertility pressure, urbanization, and demographic transition

The spatial and demographic transitions are significant to the outcome of child survival in Africa. Child mortality continues to be preeminently caused by high fertility, especially because of resource dilution, decreased maternal healthcare access, and decreased parental investment per child (12, 13). To facilitate a reinforcing feedback loop, there is a tendency to increase mortality to drive up fertility in order to create a demographic trap that makes the population health increase more slowly (25, 26). On the other hand, urbanization may facilitate the survival of children by enhancing access to healthcare, educational infrastructures, as well as sanitation amenities (27, 28). That being said, unplanned urban growth can increase the risk of being exposed to pollution, communicable disease, and overcrowded living conditions, thus undoing possible health benefits (29). The experience in African settings thus indicates that though high fertility has a constant propensity of increasing the risk of mortality, urbanization when properly controlled can reduce demographic tensions. The interaction of these aspects is one of the factors that lead to the spatial differences present in child survival throughout the continent. Accordingly, we expect fertility to exacerbate, and urbanization to alleviate, child mortality across African countries.

### 2.4 Hypothesis development

Building on the health production framework and the existing empirical literature, child mortality is viewed as an outcome shaped by economic capacity, public health investment, environmental quality, and demographic structure. Economic growth enhances household income and government fiscal space, thereby improving access to nutrition and healthcare services, while public health expenditure strengthens preventive and curative health systems. Conversely, environmental degradation and demographic pressure impose significant health burdens by increasing disease exposure and constraining household-level health investments. In the African context, these effects are expected to be heterogeneous, reflecting differences in baseline vulnerability, institutional capacity, and access to essential services across countries. Accordingly, the following hypotheses are proposed:

*H1*: Economic growth reduces child mortality in African countries.

*H2*: Higher public health expenditure reduces child mortality in African countries.

*H3*: Air pollution increases child mortality in African countries.

*H4*: Higher fertility rates increase child mortality in African countries.

*H5*: Urbanization reduces child mortality in African countries.

**Figure 1:**
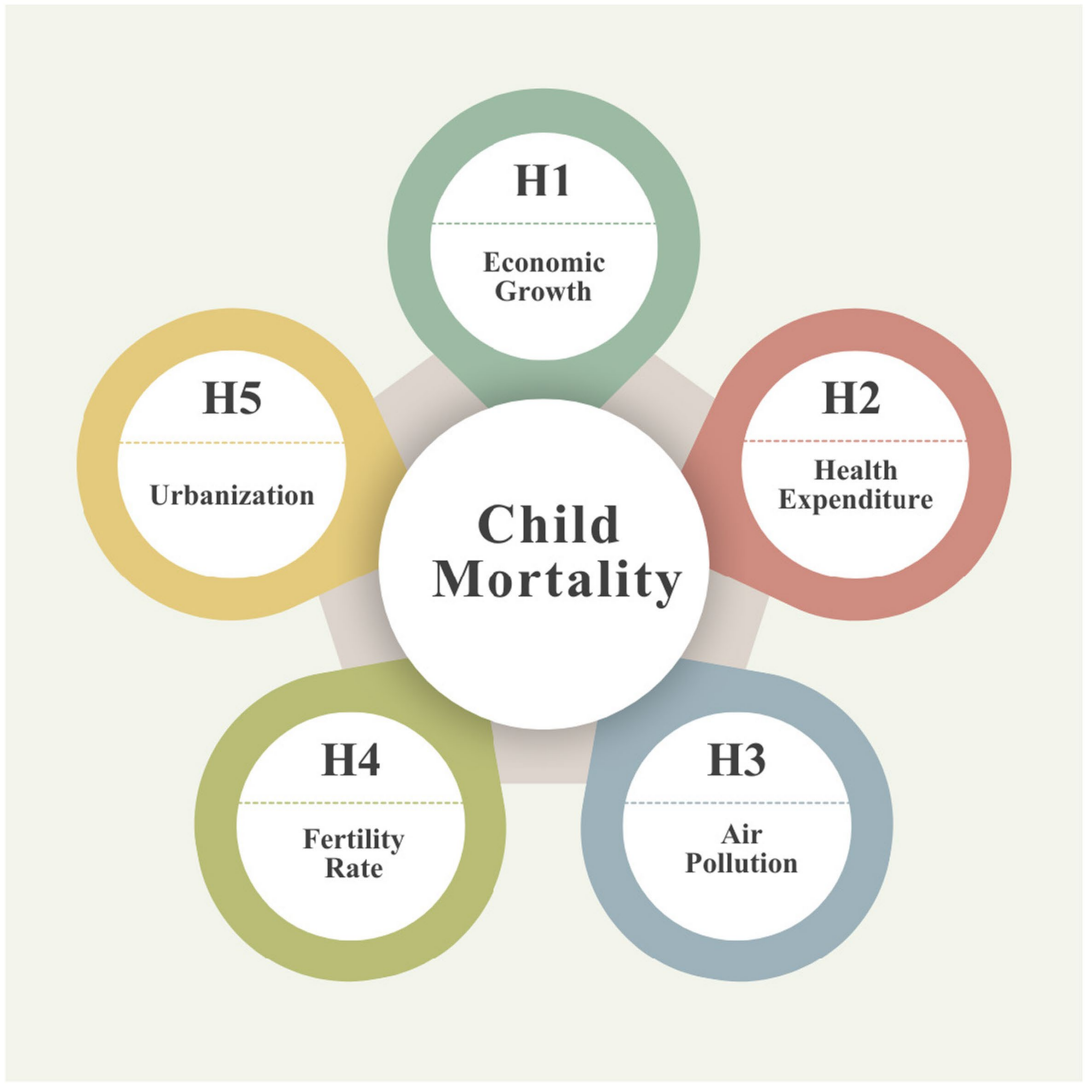
Hypothesis development of the study.

Figure 1 demonstrates hypothesis development of the study.

### 2.5 Literature gap and research contribution

Although numerous studies have examined the determinants of child mortality, most have treated economic, environmental, and demographic factors as isolated influences rather than as interacting systems. Previous research has typically emphasized income growth, fertility, or health expenditure individually, overlooking how these dimensions collectively shape child survival outcomes across the African continent. Critical gaps therefore persist in understanding the simultaneous and conditional effects of these variables, particularly in resource-constrained settings where multiple risks coexist (2, 4, 7). First, limited research has explored how economic growth and health expenditure interact under conditions of high fertility and environmental deterioration. While evidence suggests that stronger economies and increased health investments reduce mortality (6, 19), there remains uncertainty regarding whether these benefits persist when pollution and fertility pressures rise concurrently. Existing cross-country analyses often rely on mean-based models that neglect heterogeneity in mortality intensity, thereby obscuring disparities between low- and high-mortality nations (1, 15).

Second, environmental degradation, particularly air pollution, has been recognized as a critical health risk, yet few studies have integrated it with socioeconomic and demographic dynamics in the African context. Research indicates that high levels of particulate matter and carbon emissions increase child deaths (8, 10, 11), but most environmental studies remain detached from development modeling. This fragmentation prevents a thorough understanding of how pollution may offset the health gains achieved through economic growth or greater healthcare investment. Furthermore, empirical work only rarely considers the moderating role of urbanization, which can either alleviate or intensify mortality risk depending on governance and infrastructure quality (12–14). Third, comparative regional analyses often limit their focus to sub-Saharan Africa, creating a gap in continent-wide evidence that covers the collective African Union framework. As a result, there is limited understanding of how regional policy heterogeneity, economic structure, and demographic transition jointly influence child survival across Africa’s northern, western, eastern, central, and southern subregions (3, 4).

To address these limitations, the present research provides the first continent-wide empirical assessment of the joint and interactive effects of economic growth, health expenditure, urbanization, fertility, and air pollution on child mortality in 53 African Union countries from 2000 to 2024. By adopting a multidimensional perspective, this study reveals the asymmetric and conditional nature of mortality determinants that previous analyses have overlooked (2, 15). The approach demonstrates how the benefits of economic expansion and urban growth can be constrained by high fertility and environmental stress in low-income contexts. Empirically, the study contributes by employing a distribution-sensitive analytical framework that captures variation across the mortality distribution, identifying structural vulnerabilities among high-mortality countries that traditional mean-based estimations conceal (1, 4). Theoretically, it situates the child mortality debate within an integrated sustainability paradigm, linking economic prosperity, environmental health, and demographic transition, thereby expanding the scope of development and public-health literature in Africa (9, 11). Overall, this research fills a critical gap by offering a comprehensive, evidence-based understanding of how multiple development drivers interact to shape child survival outcomes. It advances the discourse on achieving Sustainable Development Goal 3.2 within Africa’s unique demographic and environmental context and underscores the necessity for coordinated African Union policies that align economic growth with environmental protection and health system resilience.

## 3 Methodology

This section highlights the research design used to investigate the determinants of child mortality in the African Union members. It also provides the model specification, sources of data, and construction of the variables, and then the econometric methods used to obtain robust estimation. This research combines second-generation panel diagnostics with the distribution-sensitive quantile method, which allows researchers to capture both the cross-country heterogeneity and the asymmetric relationships among the economic, environmental, and demographic variables that contribute to child survival.

### 3.1 Data and variables

The present study uses the concept of balanced panel data comprising 53 member countries of the African Union from 2000--2024. The data combines data on the World Development Indicators (WDI), the World Health Organization (WHO), and our World in Data so that there is consistency, coverage, and comparability of the data over time and across nations. This period was selected according to the time of the heightened health and environmental reforms in Africa, such as the Millennium Development Goals (MDGs) and the shift to the Sustainable Development Goals (SDGs). Applying this analysis to the year 2024 enables the consideration of post-pandemic recovery processes that can potentially transform the economic–health–environment nexus (2, 15).

Child mortality (CM) will be used as the dependent variable, measured as the number of deaths of children under five per 1,000 live births. It serves as a useful proxy for assessing the extent of child health and overall population well-being within a country, which has been widely used in the existing literature (30, 31). In addition, economic growth (GDP per capita), air pollution (CO emissions per capita), health spending (as a percentage of GDP), urbanization (urban population share), and the fertility rate (births per woman) are the explanatory variables. These variables are based on empirical evidence that has suggested relationships between income, pollution, demographic pressures, and mortality (7, 9, 11). The empirical setting is Africa since it has the highest rate of under-five mortality globally and is highly heterogeneous in terms of institutional, environmental, and demographic characteristics (4, 32). Table 1 summarizes the variable definitions and the source and measurement units.

**Table 1:** Description and sources of variables.

Variables	Description	Log form	Unit of measurement	Source
CM	Child mortality	LCM	Infant deaths per 1,000 live births	WHO
GDP	Economic growth	LGDP	GDP per capita current U. S. dollars	WDI
CO_2_	Air pollution	LCO_2_	Carbon dioxide emissions per capita	WDI
URP	Urban population	LURP	Percent urban population	WDI
HE	Health expenditure	LHE	Health spending as a percent of GDP	WDI
FR	Fertility rate	LFR	Fertility rate births per woman	Our world in data

### 3.2 Theoretical framework and model specification

This theoretical framework is based on the conceptual provisions of the health output (HO) model created by Grossman (33) and views health as an outcome of many other inputs, such as income, education, quality of the environment, and access to healthcare. The Grossman model initially concentrated on micro-level individual behavior, in which health is a type of capital that individuals can invest in through preventive and curative efforts. On this basis, later authors such as Majeed and Ozturk (34) and Siddique and Kiani (35) extended the HO model to macroeconomic factors in which national and environmental conditions coexist and shape the health status of societies. This paper further extends the HO framework to the African context by defining child mortality as a negative measure of health output, while population-related (fertility, urbanization) and environmental (air pollution) characteristics are treated as inputs into the health production process. The modified framework assumes that health outcomes improve with rising economic growth and with increased investment in healthcare but deteriorate under demographic pressure and environmental degradation.

Mathematically, the relationship can be expressed in Equation 1:


HO=∫(Economic,Environmental,Demographic) (1)

The extended health production relationship now can be write in Equation 2:


$$\mathrm{HO}=f(\mathrm{GDP},CO_{2},\mathrm{URP},\mathrm{HE},\mathrm{FR})\;$$ (2)

This study investigates the relationship between child mortality and a set of economic, environmental, and demographic factors across African Union member states. The model extends the macro-level health output framework to include the principal variables discussed in Section 2, namely, economic growth, health expenditure, air pollution, fertility, and urbanization. Let i = 1, …, 53 index African Union member countries and t = 2000, …, 2024 index years. The dependent variable CM_it_ denotes under-five child mortality (deaths per 1,000 live births). The regressors include income GDP_it_ (GDP per capita), air pollution CO₂_it_ (carbon dioxide emissions per capita), health expenditure HE_it_ (percent of GDP), urbanization URP_it_ (urban population share), and fertility FR_it_ (births per woman). Then Equation 3 formulated as follows:


$$\begin{array}{l} CM_{\mathrm{it}}=\beta_{0}+\beta_{1}\mathrm{GD}P_{\mathrm{it}}+\beta_{2}CO_{2_{\mathrm{it}}}+\beta_{3}\mathrm{UR}P_{\mathrm{it}}\\ +\beta_{4}HE_{\mathrm{it}}+\beta_{5}FR_{\mathrm{it}}+\varepsilon_{\mathrm{it}} \end{array}$$ (3)

Equation 4 applies a natural logarithmic transformation to all variables. Equation 4 can be written as follows:


$$\begin{array}{l} \mathrm{LC}M_{\mathrm{it}}=\beta_{0}+\beta_{1}\mathrm{LGD}P_{\mathrm{it}}+\beta_{2}\mathrm{LC}O_{2_{\mathrm{it}}}+\beta_{3}\mathrm{LUR}P_{\mathrm{it}}\\ +\beta_{4}\mathrm{LH}E_{\mathrm{it}}+\beta_{5}\mathrm{LF}R_{\mathrm{it}}+\varepsilon_{\mathrm{it}} \end{array}$$ (4)

Where, $\beta_{1}$ to $\beta_{5}$ denote coefficients of explanatory variables, $\varepsilon_{\mathrm{it}}$ used as error term and $\beta_{0}$ shows intercept. Taking logarithms allows the coefficients to be interpreted as elasticities, indicating the percentage change in child mortality resulting from a one-percent change in each explanatory variable. The log-linear specification also helps stabilize variance, reduce heteroskedasticity, and better capture nonlinear relationships among variables across diverse African economies.

Table 2 clarifies the theoretical pathway linking macroeconomic, environmental and demographic factors to child mortality, and directly supports the empirical specification adopted in the study.

**Table 2:** Theoretical framework and expected direction of effects.

Variable	Expected effect
LGDP	↑ → Child mortality ↓ (higher income improves health access, nutrition, sanitation)
LHE	↑ → Child mortality ↓ (public health investment enhances service delivery and outcomes)
LFR	↑ → Child mortality ↑ (fertility increases resource dilution and maternal burden)
LURP	↑ → Child mortality ↓ (urbanization increases service access, medical facilities, sanitation)
LCO₂	↑ → Child mortality ↑ (pollution damages respiratory and neonatal health)

### 3.3 Empirical strategy

A series of diagnostic tests was applied before the actual implementation of the main estimations to ensure that the econometric model would be valid and robust. The variance inflation factor (VIF) was initially used to test the possibility of multicollinearity existing between the explanatory variables and thus ensure that the correlations were within the critical range and therefore did not affect the estimates of the coefficients. Subsequently, cross-sectional dependence (CSD) was tested via the Pesaran (36) test, which revealed intercountry connections due to shared regional shocks such as health crises and economic spillovers. Pesaran and Yamagata (37) proposed a slope heterogeneity test that was then utilized to test structural differences among countries. The unit root tests Levin–Lin–Chu (LLC), cross-sectional Im-Pesaran–Shin (CIPS), and augmented Dickey–Fuller (ADF) were used to determine the order of integration (38–40). These gave a combination of I(0) and I(1) series. The relationships of variables in the long run were also established with the help of Westerlund and Edgerton (41), Pedroni (42), and Kao (43) cointegration tests.

**Figure 2:**
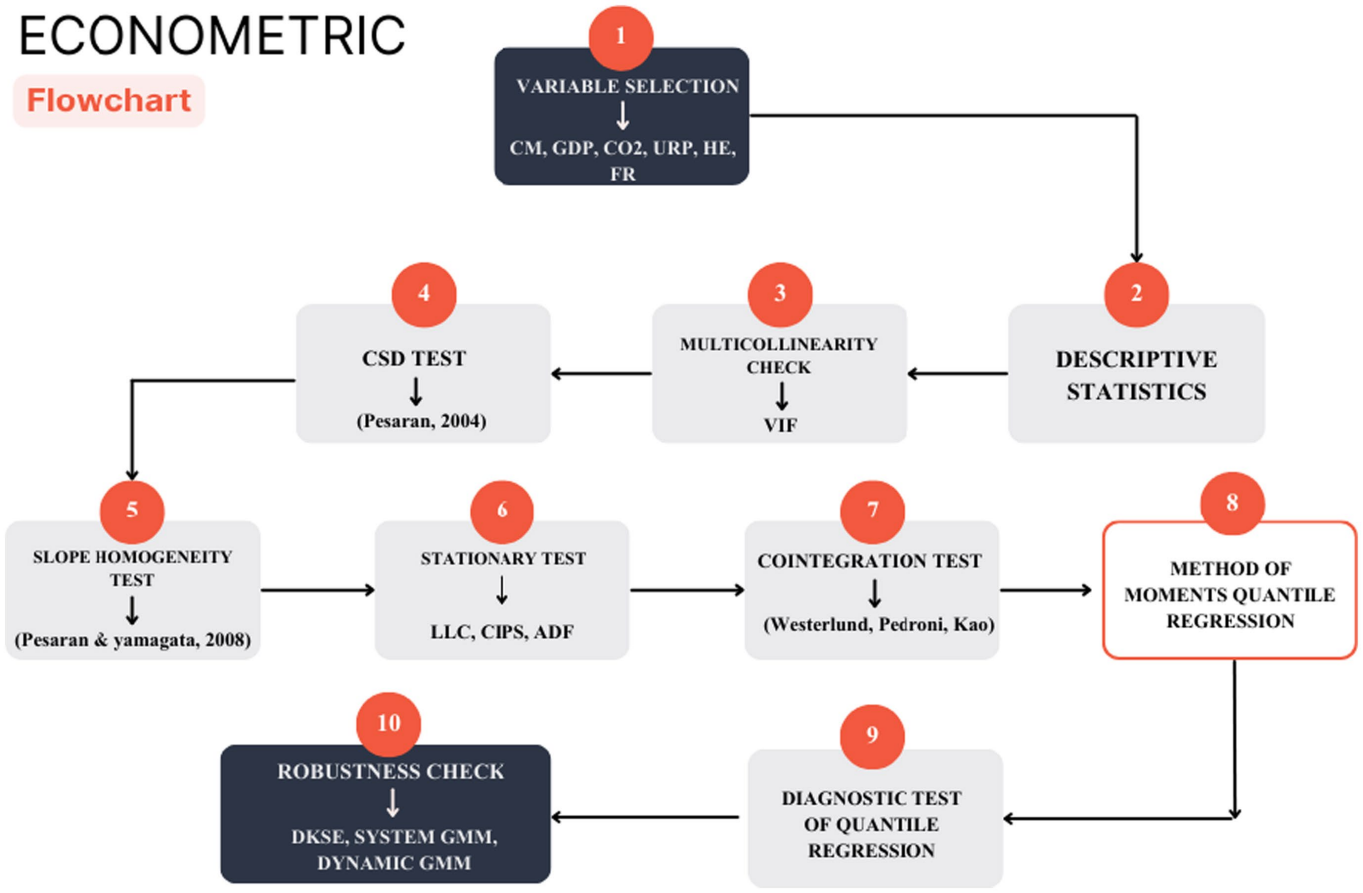
Flow of empirical estimation.

To approximate the heterogeneous determinants of child mortality, the method of moments quantile regression (MMQR) by Machado and Silva (17) is used in the present study. The MMQR records distributional heterogeneity in that it permits the variation of coefficients across quantiles, with more detailed insight into how economic, environmental, and demographic variables influence mortality at lower and higher levels of burden. Complementary estimators were employed to prove the strength of the MMQR estimates. The Driscoll–Kraay standard error (DKSE) methodology eliminates cross-sectional dependence, heteroskedasticity, and serial correlation, which guarantees unbiased standard errors. Moreover, the endogeneity, which occurs as a result of reverse causality between the economic variables and health variables, was controlled with the aid of system GMM and difference GMM estimators. These models are able to guarantee consistent and efficient estimation by employing internal instruments (lagged regressors) and specification tests such as AR (1), AR (2), Sargan and Hansen. The consistency of the findings of the MMQR, DKSE and GMM approaches corroborates the reliability and stability of the findings in explaining child mortality in African countries (Figure 2).

## 4 Results and discussion

To approximate the heterogeneous determinants of child mortality, the MMQR by Machado and Silva (17) is used in the present study. The MMQR records distributional heterogeneity in that it permits the variation of coefficients across quantiles, with more detailed insight into how economic, environmental, and demographic variables influence mortality at lower and higher levels of burden. Complementary estimators were employed to prove the strength of the MMQR estimates. The Driscoll–Kraay standard error (DKSE) methodology eliminates cross-sectional dependence, heteroskedasticity, and serial correlation, which guarantees unbiased standard errors. Moreover, the endogeneity, which occurs as a result of reverse causality between the economic variables and health variables, was controlled with the aid of system GMM and difference GMM estimators. These models are able to guarantee consistent and efficient estimation by employing internal instruments (lagged regressors) and specification tests such as AR (1), AR (2), Sargan and Hansen. The consistency of the findings of the MMQR, DKSE and GMM approaches corroborates the reliability and stability of the findings in explaining child mortality in African countries (Table 3).

**Table 3:** Descriptive statistics.

Variable	Obs	Mean	Std. dev.	Min	Max
LCM	1,325	3.831	0.565	2.079	4.927
LGDP	1,325	7.141	1.062	4.697	9.860
LCO_2_	1,325	−0.802	1.423	−3.912	2.554
LURP	1,325	3.677	0.465	2.110	4.514
LHE	1,325	1.545	0.450	−1.017	3.540
LFR	1,325	1.457	0.355	0.278	2.058

The initial diagnostic findings that confirm the strength of the panel dataset are summarized in Tables 4–6. The findings of the variance inflation factor (VIF) in Table 4 indicate that all the variables used have a coefficient that is far less than the cutoff point of 10, which confirms that there is no multicollinearity and that the estimation of the coefficient is trustworthy. Table 5 Cross-sectional dependence (CSD) results reported by Pesaran (36). The CD statistic rejects the null hypothesis of independence at the 1 percent level. This implies the existence of intercountry linkages among African countries, which are propelled by common health, economic, and environmental shocks. Similarly, the slope homogeneity test in Table 6, according to Pesaran and Yamagata (37), rejects the fact that the number of parameters used is the same, which is confirmed by the fact that the relationships between explanatory variables and child mortality vary across countries.

**Table 4:** Multicollinearity test.

Variable	VIF	1/VIF
LGDP	2.77	0.361
LCO_2_	3.00	0.333
LURP	0.99	1.010
LHE	0.63	1.587
LFR	1.44	0.694
Mean VIF	1.77	

**Table 5:** CSD test.

Variable	CD- stat	*p* value
LCM	162.552	0.000***
LGDP	144.602	0.000***
LCO_2_	36.053	0.000***
LURP	145.695	0.000***
LHE	4.223	0.000***
LFR	136.314	0.000***

***p < 0.01.

**Table 6:** Slope homogeneity test.

Test	Delta	*p*-value
	38.676	0.000
	45.580	0.000
Adj.		

Table 7 summarizes the results of the panel unit root tests, which were carried out under the Levin–Lin–Chu (2002) approach, cross-sectional Im–Pesaran–Shin [CIPS, (40)] approach, and augmented Dickey–Fuller (38) approach. The results suggest that the variables have mixed orders, I(0) and I(1), meaning that some of them remain stationary at level and others at first differencing. This combination verifies the suitability of estimators capable of accommodating such integration characteristics, specifically, the method of moments quantile regression (MMQR) and GMM models.

**Table 7:** Panel unit root test.

Variable	LLC		CIPS		ADF		Order
	I(0)	I(1)	I(0)	I(1)	I(0)	I(1)	
LCM	−5.473***	−14.144***	−2.011	−4.012***	181.202	281.633***	I(1)
LGDP	−12.898***	−25.015***	−2.221***	−4.477***	201.516***	511.699***	I(0)
LCO_2_	−10.696***	−27.484***	−2.517***	−4.715***	130.836*	611.781***	I(0)
LURP	−10.945***	−0.619	−1.453	−1.312	509.103***	298.361***	I(1)
LHE	−9.782*	−22.237***	−1.623	−4.420***	121.395	485.365***	I(1)
LFR	0.212	−13.897***	−1.591	−2.559***	40.381	228.589***	I(1)

***p < 0.01; **p < 0.05; *p < 0.1.

The results of the panel cointegration tests conducted by Westerlund and Edgerton (41), Pedroni (42), and Kao (43) to investigate the presence of long-term relationships between child mortality, economic growth, air pollution, urbanization, health expenditure and the fertility rate are presented in Table 8. In all the specifications, including those with no deterministic trends, the null hypothesis of no cointegration is rejected at the 1 percent level, which attests to a long-term stable equilibrium between the variables. The relevance of the variance ratio statistics and the Phillips–Perron statistics proves that these socioeconomic and environmental indicators change in the same direction over time. This fact confirms the theoretical hypothesis that the negative influences of pollution and fertility are neutralized by the positive impacts of the sustained growth of income, investment in healthcare, and urbanization.

**Table 8:** Panel cointegration test.

Test		Without trend		with trend (all-panels)	
		Statistics	*p* value	Statistics	*p* value
Westerlund	Variance Ratio	−2.482	0.006	2.516	0.005

Test		Without trend		with trend	
		Statistics	*p* value	Statistics	*p* value
Pedroni	Modified Phillips-Perron t	5.754	0.000	6.210	0.000
	Phillips-Perron t	−3.843	0.000	−6.910	0.000
	Augmented Dickey-Fuller t	−1.589	0.056	−4.958	0.000

Test		Statistics	*p* value
Kao	Modified Dickey-Fuller t	2.986	0.001
	Dickey-Fuller t	1.093	0.137
	Augmented Dickey-Fuller t	0.633	0.263
	Unadjusted modified Dickey-Fuller t	3.552	0.000
	Unadjusted Dickey-Fuller t	1.725	0.042

The consistently negative impact of economic growth on child mortality across all quantiles, as evidenced by the MMQR estimates reported in Table 9, indicates a structurally robust income–health relationship in Africa. Importantly, the increasing magnitude of the income effect toward the upper quantiles of the mortality distribution suggests that economic growth yields disproportionately larger survival gains in high-mortality settings. This pattern reflects a non-linear health response whereby income improvements in severely deprived environments translate into substantial gains in nutrition, disease prevention, and access to basic healthcare services, thereby enhancing household-level health resilience. This finding is consistent with Haque and Farid (19) and Rajapakse et al. (20), who document stronger mortality reductions from income growth in low-income contexts, as well as Yeboah et al. (21), who emphasize the role of economic expansion in improving healthcare affordability in Sub-Saharan Africa. However, the heterogeneous strength of the income effect across quantiles also resonates with the concerns raised by Freeman et al. (44), suggesting that unequal growth distribution may partially offset health gains in relatively better-performing settings. By explicitly capturing these distributional differences using the MMQR framework, the results in Table 9 extend the existing literature beyond average effects and confirm Hypothesis H1, highlighting that economic growth is most effective in reducing child mortality where baseline vulnerability is highest.

**Table 9:** Method of moments quantile regression.

Variables	Location	Scale	(1) Q0.05	(2) Q0.25	(3) Q0.50	(4) Q0.75	(5) Q0.95
LGDP	−0.0913*** (0.0198)	−0.0071* (0.0120)	−0.0777*** (0.0283)	−0.0851*** (0.0212)	−0.0971*** (0.0231)	−0.111*** (0.0351)	−0.107*** (0.0343)
LCO₂	0.0095* (0.0154)	0.0408*** (0.0093)	0.0680*** (0.0223)	0.0257* (0.0165)	0.0427** (0.0181)	0.0948*** (0.0272)	0.0969*** (0.0266)
LURP	−0.0684*** (0.0249)	−0.0204* (0.0151)	−0.0297* (0.0356)	−0.0508* (0.0266)	−0.0851*** (0.0291)	−0.109** (0.0408)	−0.112*** (0.0431)
LHE	−0.0451* (0.0237)	−0.0675*** (0.0143)	−0.0833** (0.0342)	−0.0132* (0.0253)	−0.100*** (0.0278)	−0.186*** (0.0415)	−0.190*** (0.0408)
LFR	1.1851*** (0.0456)	1.6720*** (0.0276)	1.1981*** (0.0651)	1.1912*** (0.0488)	1.1793*** (0.0533)	1.1581*** (0.0804)	1.1701*** (0.0791)
Constant	2.4421*** (0.1991)	0.1610* (0.1202)	2.1360** (0.2841)	2.3031*** (0.2121)	2.5741** (0.2322)	2.8011*** (0.3511)	2.7880*** (0.344)0

Standard errors in parentheses. ***p < 0.01; **p < 0.05; *p < 0.1.

The MMQR estimates reported in Table 9 reveal a consistently negative and statistically significant relationship between public health expenditure and child mortality across all quantiles, underscoring the central role of health investment in improving child survival in Africa. Notably, the magnitude of the effect intensifies toward the upper quantiles of the mortality distribution, indicating that increased health spending delivers substantially larger benefits in high-mortality settings. This distributional pattern suggests that investments in healthcare infrastructure, immunization coverage, maternal services, and primary care systems are particularly effective in contexts characterized by limited baseline access to health services, thereby narrowing mortality disparities. These findings align with Betila (22) and Wassou et al. (45), who highlight the life-extending effects of public health expenditure in Africa, especially when supported by ICT-enabled health management systems. Consistent evidence is also provided by Owusu et al. (23) and Ayipe and Tanko (46), who show that targeted public spending reduces child mortality when institutional effectiveness ensures efficient resource allocation. However, the heterogeneity observed across quantiles also resonates with the concerns raised by Kouadio and Mom (24), suggesting that administrative inefficiencies can attenuate the mortality-reducing impact of health budgets in relatively better-performing settings. By capturing these asymmetric effects, the results in Table 9 extend existing mean-based evidence and strongly support Hypothesis H2, emphasizing that health expenditure is most impactful in reducing child mortality where vulnerability is greatest.

The MMQR results demonstrate a consistently positive and statistically significant association between air pollution and child mortality across all quantiles, highlighting the pervasive health burden of environmental degradation in African countries. Notably, the strengthening of the pollution coefficient toward the upper quantiles of the mortality distribution indicates that the adverse health effects of pollution are disproportionately concentrated in high-mortality settings. This pattern reflects heightened vulnerability in environments where children already face compounded risks from poor nutrition, limited healthcare access, and inadequate environmental protection, thereby amplifying the respiratory, immunological, and neonatal impacts of air pollution. These findings are consistent with Karimi and Shokrinezhad (10), Wang et al. (11), and Kelly and Wassou (47), who document elevated infant and child mortality linked to particulate exposure in developing economies. Supporting evidence from Adeleye et al. (8) further emphasizes that industrial emissions and fossil-fuel dependence pose direct threats to child health in Africa. At the same time, the heterogeneous magnitude of the estimated effects aligns with Hassan and Murad (9), suggesting that effective environmental regulation can partially mitigate pollution-related mortality in relatively better-performing contexts. By uncovering these asymmetric pollution effects across the mortality distribution, the MMQR estimates extend existing mean-based evidence and provide strong support for Hypothesis H3, underscoring that environmental degradation is most lethal where baseline child health vulnerability is highest.

The MMQR estimates reported show a consistently positive and statistically significant relationship between fertility and child mortality across all quantiles, underscoring the dominant demographic pressure exerted by high fertility in African countries. The large and elastic coefficients observed throughout the mortality distribution indicate that higher fertility rates substantially increase child mortality risk by intensifying resource dilution, constraining household health investment, and weakening maternal care and supervision. Importantly, the persistence of this effect across quantiles suggests that fertility-related health burdens are not confined to extreme mortality settings but operate as a structural constraint on child survival across diverse demographic contexts. These findings are consistent with Haq et al. (13) and Angko et al. (12), who emphasize that high fertility limits healthcare utilization and amplifies child vulnerability in resource-constrained households. Complementary evidence from Jayathilaka et al. (14) further supports the mortality-reducing role of fertility transitions in developing economies. At the same time, the results resonate with the bidirectional demographic dynamics discussed by Fareed et al. (25), indicating that mortality decline may also induce fertility reduction over time. By explicitly revealing the magnitude and distributional persistence of fertility effects, the MMQR results in Table 9 extend prior evidence and strongly confirm Hypothesis H4, highlighting fertility as a critical driver of child mortality and reinforcing the importance of integrated family planning and maternal health interventions in Africa.

The MMQR estimates indicate a consistently negative and statistically significant relationship between urbanization and child mortality across all quantiles, suggesting that urban expansion plays a protective role in child survival in African countries. The increasing magnitude of the urbanization effect toward higher mortality quantiles implies that the health benefits of urban living are particularly pronounced in high-mortality settings. This distributional pattern reflects the concentration of healthcare facilities, educational opportunities, sanitation infrastructure, and reliable electricity supply in urban areas, which collectively enhance access to preventive and curative health services and strengthen child health resilience. These findings are consistent with Oyekale and Maselwa (28) and Asumadu-Sarkodie and Owusu (27), who document substantial reductions in under-five mortality associated with improved urban infrastructure and service delivery. At the same time, the heterogeneous effects observed across quantiles resonate with the concerns raised by Bidzha et al. (29), who caution that unplanned urbanization may expose vulnerable populations to pollution and communicable diseases, potentially offsetting health gains. Nevertheless, the overall negative association underscores that well-managed urbanization serves as an effective channel for reducing child mortality, thereby confirming Hypothesis H5 and highlighting the importance of planned urban growth in improving child health outcomes across Africa.

Table 10 presents a summary of the diagnostic checks used to validate the MMQR model. The tests of the residual reveal the nonexistence of heteroskedasticity, serial correlation, and specification bias and that the model dynamics are well-behaved. The quantile-process stability and the pseudo-R indicate the reliability of the models in explaining distributional variation in child mortality. The adequacy of the models is also supported by the Durbin–Watson and Jarque–Bera normality outcomes. All these diagnostics certify that MMQR estimates are very strong, effective, and free of endogeneity distortion, thus confirming that the effects of economic growth, health expenditure, fertility, urbanization and pollution are statistically sound and policy-relevant in Africa.

**Table 10:** Diagnostic test of quantile regression.

Quantile	Wald *χ*^2^ statistic	*p* value	Pseudo *R*^2^
Q0.05	41.26	0.000	0.112
Q0.25	46.78	0.000	0.154
Q0.50	52.94	0.000	0.198
Q0.75	58.33	0.000	0.241
Q0.95	63.71	0.000	0.265

Table 11 shows consistency findings based on Driscoll-Kraay (DKSE), system GMM and dynamic GMM estimators to confirm the consistency of the MMQR results. The findings affirm that economic growth, expenditures on health, and urbanization have substantial negative effects on child mortality, but air pollution and fertility have positive effects. The sense and value of the coefficients are the same among all estimators, which adds reliability to the robustness of the models. Diagnostic statistics such as AR(1) and AR(2) lack serial correlation, whereas the Sargan and Hansen tests provide evidence of the validity of the instruments. The methods are efficient in solving for endogeneity, heteroskedasticity, and cross-sectional dependence to obtain undiscriminate and efficient parameters. The matching of the DKSE and GMM results improves the level of self-belief in the empirical reliability and stability of the determinants of child mortality identified in African countries.

**Table 11:** Robustness check (DKSE, system GMM, dynamic GMM).

Variables	(1) DKSE	(3) system GMM	(4) dynamic GMM
L. LHE			0.3120*** (0.0721)
LGDP	−0.0911** (0.0381)	−0.0850** (0.0340)	−0.0711** (0.0330)
LCO₂	0.0095* (0.0056)	0.0123** (0.0052)	0.0114** (0.0050)
LURP	−0.0684*** (0.0201)	−0.0715*** (0.0213)	−0.0698*** (0.0207)
LHE	−0.0451** (0.0189)	−0.0528** (0.0224)	−0.0502** (0.0219)
LFR	1.1851*** (0.0412)	1.1621*** (0.0386)	1.1490*** (0.0368)
Constant	2.4421*** (0.5220)	2.3951*** (0.4010)	2.3621*** (0.387-)
AR(1) *p* value		0.0311	0.0201
AR (2) *p* value		0.4820	0.5160
Sargan test *p* value		0.5171	0.3430
Hansen test *p* value		0.6411	0.5140

## 5 Discussion and conclusion

### 5.1 Theoretical implications

The findings of this study provide substantial theoretical contributions to the disciplines of health economics, sustainable development, and demographic studies. Anchored in the health output framework of Grossman (33), the results reaffirm that health outcomes are not the result of a single economic or social determinant but rather emerge from the interaction of multiple economic, environmental, and demographic forces. In this sense, health is best viewed as a multidimensional stock of human capital produced jointly by household behavior, public investment, and environmental conditions (1, 4).

By extending the Grossman model to the macro level, this research conceptualizes child mortality as a negative outcome of health production—reflecting the structural capacity of nations to sustain human development. Unlike traditional growth–health frameworks that predominantly rely on income-driven explanations of health, the present study integrates economic growth, environmental quality, fertility dynamics, health expenditure, and urbanization into a single analytical system. This multidimensional framework aligns with contemporary sustainability theories that emphasize the interdependence of economic prosperity, environmental stewardship, and institutional performance (9, 15).

The evidence that fertility and pollution accelerate child mortality even under conditions of economic expansion challenges traditional growth-centric perspectives (6, 11). Such findings suggest that income gains alone are insufficient for sustainable health improvements if environmental degradation and high fertility persist. The results thus support the emerging consensus that economic progress can only translate into better population health when accompanied by demographic transition and ecological preservation (2, 7). Theoretically, this implies a composite model of health production in which environmental stressors and demographic pressures act as counterforces to the benefits of growth and health investment.

Furthermore, the quantile-based estimation reveals critical distributional heterogeneity in the determinants of child mortality. The study finds that the effects of economic growth, healthcare expenditure, fertility, and air pollution vary systematically across country-specific mortality levels. This variation indicates that the health production frontier is non-linear and conditional on structural and institutional thresholds, a notion consistent with Zakaria et al. (4) and Gayawan et al. (15). In practical terms, the marginal returns to income or health spending are greater in high-mortality countries with weaker institutions and higher demographic burdens. This insight extends Grossman’s original formulation by introducing a threshold-sensitive dimension to the macro-economic determinants of health. Hence, this study reaffirms that achieving sustained improvements in child health requires an integrated framework that simultaneously advances economic capacity, strengthens institutional and demographic systems, and mitigates environmental degradation, thus redefining health output as a multidimensional outcome of sustainable development.

### 5.2 Policy and managerial recommendations

The results have important managerial and policy implications for governments, regional bodies, and development partners. Given the heterogeneity revealed through the findings of the present study, interventions should be differentiated across country groups. For high-mortality, high-fertility countries, where fertility pressures and environmental stressors strongly dampen the benefits of income expansion, policy priority should be directed toward rapid fertility decline via universal reproductive health services, expanded family planning, and girls’ secondary education, consistent with the demographic transition pathway outlined by Amegbor and Addae (2) and Rahman and Alam (7). Strengthening primary healthcare, maternal-child services, and immunization networks is particularly relevant in fragile settings where health spending shows the highest marginal return, as also evidenced by Rahman et al. (1). In these contexts, performance-linked health financing and conditional public transfers may accelerate the mortality response.

For high-pollution emerging economies, where income and urbanization contribute to mortality reduction yet are offset by environmental degradation, coordinated energy–environment governance is essential. Investments should prioritize clean household energy, industrial emission control, and air-quality monitoring, aligning with sustainability arguments in Gayawan et al. (15) and Hassan and Murad (9). Ministries of health, energy, and environment must jointly design pollution-responsive child health interventions that expand access to clean energy technologies while scaling neonatal and respiratory care. Meanwhile, lower-mortality transitioning countries, where demographic burdens are declining and health systems expanding, require policies that consolidate gains. Here, the focus should shift toward surveillance-based mortality tracking, urban planning, and digital health systems that maintain downward mortality trends, echoing institutional and threshold effects highlighted by Zakaria et al. (4). At the regional level, the African Union, AfDB, and UNECA should adopt monitoring frameworks under the Africa Health Strategy (2023–2030) to classify countries into the three categories and tailor financing windows accordingly. South–South cooperation with emerging economies in Asia and Latin America can further support technology transfer in telehealth, renewable energy, and demographic governance. Local implementation should involve civil-society actors, youth groups, and community health workers to strengthen accountability, enhance reproductive autonomy, and reinforce public awareness around environmental risks. Through such interventions, progress toward SDG 3.2 becomes more feasible and equitable across Africa’s heterogeneous mortality landscape.

### 5.3 Limitations and future research direction

Although the present study provides robust empirical and theoretical insights into the determinants of child mortality in Africa, several limitations should be acknowledged. First, while the use of MMQR and GMM estimations effectively addresses issues of endogeneity, heterogeneity, and distributional asymmetry, the approach remains fundamentally linear and may not fully capture complex threshold, feedback, or spatial spillover effects inherent in the development–health–environment nexus. Future studies should therefore incorporate dynamic spatial econometric frameworks or nonlinear models, such as panel threshold regressions, Bayesian hierarchical models, or machine-learning algorithms, to account for multidirectional causality among key variables like fertility, pollution, and income.

Second, the study period (2000–2024) reflects the contemporary era of health and environmental reforms in Africa but does not include earlier decades of post-independence development, structural adjustment, and pre-MDG health transitions. Historical data covering the 1970s through the 1990s could enable researchers to trace the long-term evolution of health systems and socioeconomic disparities that laid the foundations for present-day child mortality trends. Incorporating such datasets would allow for longitudinal analyses that capture the continuity and structural transformation of African health and environmental policies over time. Third, the study’s cross-national analytical design, although suitable for capturing general patterns across 53 African Union countries, may overlook unique contextual conditions at the sub-regional, national, and local levels. The contextual specificity of mortality determinants, shaped by political stability, cultural norms, and resource endowments, requires more nuanced country- or community-specific analyses. Future research could therefore focus on case studies or mixed-methods approaches that combine econometric modeling with field surveys, interviews, or administrative health data to better understand the interplay of policies and behavioral dynamics at the micro level.

Finally, comparative evidence across regions beyond Africa—such as Asia, Latin America, or the Middle East—would enhance the external validity and generalizability of the results. Cross-regional studies could help assess whether the identified distributional heterogeneities in mortality determinants are unique to Africa’s demographic and environmental conditions or represent a broader developing-world phenomenon. Overall, future research should aim to integrate richer, multidimensional datasets with innovative analytical tools to reveal the complex socio-environmental, institutional, and policy mechanisms that shape child health. Such methodological and contextual extensions would deepen the understanding of Africa’s mortality dynamics and provide more tailored policy insights supporting the Sustainable Development Goals, particularly SDG 3.2, in diverse development contexts.

## Data Availability

The original contributions presented in the study are included in the article/supplementary material, further inquiries can be directed to the corresponding author/s.
